# The Importance of Secretor-Status in Norovirus Infection Following Allogeneic Hematopoietic Stem Cell Transplantation

**DOI:** 10.3390/v14071350

**Published:** 2022-06-21

**Authors:** Lisa Swartling, Elda Sparrelid, Per Ljungman, Ksenia Boriskina, Davide Valentini, Lennart Svensson, Johan Nordgren

**Affiliations:** 1 Department of Infectious Diseases, Karolinska University Hospital, 141 86 Stockholm, Sweden; 2 Division of Infectious Diseases, Department of Medicine Huddinge, Karolinska Institutet, 141 86 Stockholm, Sweden; elda.sparrelid@regionstockholm.se; 3 Department of Cellular Therapy and Allogeneic Stem Cell Transplantation (CAST), Karolinska University Hospital Huddinge, Karolinska Comprehensive Cancer Center, 141 86 Stockholm, Sweden; per.ljungman@ki.se (P.L.); ksenia.boriskina@regionstockholm.se (K.B.); davide.valentini@regionstockholm.se (D.V.); 4 Division of Hematology, Department of Medicine Huddinge, Karolinska Institutet, 141 86 Stockholm, Sweden; 5 Division of Molecular Medicine and Virology, Department of Biomedical and Clinical Sciences, Linköping University, 58183 Linköping, Sweden; lennart.t.svensson@liu.se (L.S.); johan.nordgren@liu.se (J.N.); 6 Division of Infectious Diseases, Department of Medicine Solna, Karolinska Institute, 17177 Stockholm, Sweden

**Keywords:** norovirus, hematopoietic stem cell transplantation, immunocompromised, susceptibility, secretor status, genotype, severity

## Abstract

*Background*. Human secretor-status is a strong susceptibility factor for norovirus infection in immunocompetent people. The predominant norovirus genotype GII.4 almost exclusively infects secretors and is also associated with more severe symptoms. However, it is not known to what extent this also applies to immunocompromised individuals. Our objective was to determine the importance of secretor-status and norovirus genotype for the susceptibility and/or the clinical course of norovirus infection in allogeneic hematopoietic stem cell transplant (HCT) patients. *Methods*: This was a retrospective study of 89 HCT patients diagnosed with norovirus infection. Secretor-status and norovirus genotype were determined using stored extracted DNA or blood (*n* = 89) and fecal samples (*n* = 22), respectively. *Results*: Seven of eighty-nine (8%) of the patients were secretor-negative, a small proportion compared to the expected rate of at least 20% non-secretors in the general Swedish population. Among the genotyped samples, norovirus genotype GII.4 was predominant (*n* = 12) and only detected in secretor-positive individuals. Patients with norovirus GII.4 had a median symptom duration of 36 (3–681) days compared to 15 (1–94) days in patients infected with other norovirus genotypes (*n* = 10, *p* = 0.1). *Conclusions*: The results suggest that secretor-status affects the susceptibility to norovirus infection even when the immune system is severely compromised. The norovirus genotype may also be a risk factor for chronic norovirus symptoms in immunocompromised patients.

## 1. Introduction

Norovirus is the leading cause of viral gastroenteritis worldwide [1]. Norovirus normally causes short-term diarrhea and vomiting, but can give rise to chronic and/or severe gastroenteritis in immunocompromised individuals [2,3,4,5]. 

The ability of noroviruses to infect humans strongly depends on the presence of different histo-blood group antigens (HBGAs) on gut mucosal surfaces [6,7,8]. In the general Swedish population, 21–28% do not express certain HBGAs due to inactivating mutations in the α1,2-fucosyltransferase 2 (FUT2) gene [9,10]. These individuals are termed secretor-negative and are, with few exceptions, protected against the most common norovirus genotype (GII.4) [11]. It is not known to what extent secretor-status is also associated with the susceptibility to norovirus infection, or severity of norovirus symptoms, in immunocompromised individuals.

Norovirus GII.4 has been the predominant genotype for many years, and remains the most prevalent genotype in Sweden, although recently other norovirus genotypes have emerged as predominant in some other countries [12,13]. In immunocompetent individuals, norovirus GII.4 has been associated with a more severe clinical course than other genotypes [14,15,16]. There are no reports of the clinical picture in relation to norovirus genotypes in immunocompromised patients.

The aim of the present study was to determine the importance of secretor-status for susceptibility and the clinical course of norovirus infection in allogeneic hematopoietic stem cell transplant (HCT) patients. Our hypothesis was that secretor-negative genotype confers resistance to norovirus GII.4, and/or contributes to a mild or asymptomatic course of norovirus infection in HCT patients. An additional aim was to investigate if the norovirus genotype affects the clinical course of norovirus infection following HCT.

## 2. Materials and Methods

### 2.1. Study Design

Patients who had undergone HCT 2006–2016 at Karolinska University Hospital, Sweden, and had been diagnosed with norovirus infection were included in the study. Altogether 89 HCT patients with norovirus were identified. Sixty-three of the patients had been included in a previous norovirus study at the center [17]. The conditioning regimens and graft-versus-host disease (GVHD) prophylaxis have been described previously [18,19]. No regular screening for gastrointestinal pathogens was conducted, but prompt testing of patients with gastrointestinal symptoms was standard. The routines for microbiological testing of fecal samples included polymerase chain reaction (PCR) for noro-, sapo-, rota- and adenovirus and detection of *Clostridium difficile* toxin, and in cases of persistent diarrhea, culture for *Salmonella*, *Shigella*, *Campylobacter* and *Yersinia* and microscopy or PCR for parasite detection was performed. Norovirus and rotavirus could also be identified in vomit samples by PCR. If gastrointestinal GVHD was suspected, endoscopy was performed. Secretor-status was determined in all patients.

Clinical data were retrieved from the electronic medical records. The duration of norovirus symptoms was defined as the number of days of diarrhea and/or vomiting with the concomitant detection of norovirus, and symptoms lasting > 30 days were defined as chronic. If the norovirus infection was diagnosed before the transplantation and ongoing at the time of HCT, only days of symptoms from the time of HCT were included. Other gastrointestinal infections diagnosed during symptoms of norovirus infection were also registered. One transplant specialist re-evaluated all patients for the occurrence of active GVHD and/or mucositis during symptoms of norovirus infection. Acute and chronic GVHD were graded according to standard criteria [20,21]. The study was approved by the Regional Ethical Board of Stockholm (2017/1508-31/2).

### 2.2. Detection of Norovirus in Fecal or Vomit Samples

During 2006–2007, norovirus detection was performed using two different endpoint PCR methods targeting the polymerase gene. In one method, forward primer 5′-GCA GGT ATT TTT ACG TGC CCA-3′ (BJCV-1S) and reverse primer 5′-CGA CGC CAT CTT CAT TCA CAA A-3′ (BJCV-2AS) was used. The other method was previously presented by Lindell et al. [22]. From 2008, norovirus was detected using two real-time PCR methods based on a slight modification of the assay published by Kageyama et al. [23], targeting the ORF1-ORF2 junction. From 2014, a direct PCR (GeneXpert DX System Operators Manual, Software Version 4.0, Cepheid Xpert^®^ Norovirus, Ref GXNOV-CE-10, March 2014, Cepheid, Sunnyvale, CA, United States) was implemented. All methods provided information on the norovirus genogroup (I or II). The cycle threshold (Ct), a value from the first fecal sample (at the time of diagnosis) was obtained from the database of the laboratory and was available for samples that had been tested with the three PCR methods used in 2008 and later (76/89 samples).

### 2.3. Sequencing and Phylogenetic Genotyping of Norovirus

In patients with available fecal samples, sequencing and phylogenetic analysis were performed, as previously described [24,25,26]. Details of the methods used are outlined in the supplementary material.

### 2.4. Determining the Secretor-Status of the HCT Recipients

To determine the secretor-status, we used samples collected at the time of HCT, either stored extracted DNA or whole blood from which DNA was extracted, as described previously by Bucardo et al. [27]. The extracted DNA was analyzed for the *FUT2* G428A (rs601338) nonsense single nucleotide polymorphism (SNP) using the TaqMan^®^ SNP Genotyping Assay (Applied Biosystems, Carlsbad, CA, USA) [27].

### 2.5. Statistics

For categorical variables, Fisher’s exact test was used for testing differences between proportions in secretor-positive vs. secretor-negative and norovirus GII.4 vs. norovirus non-GII.4. For the same group comparisons, differences between median values of numerical variables were tested using Mood’s test. The logistic regression method was used for analyzing factors related to chronic symptoms (>30 days) of norovirus infection. Factors with a *p*-value ≤ 0.20 in the univariate analysis were introduced into the stepwise selection multivariate analysis. The analyses were performed using R software (R Core Team, Vienna, Austria).

## 3. Results

Eighty-nine HCT patients with norovirus infection were identified; 42/89 (47%) were children < 18 years. In total, 886 HCTs were conducted during the study period. Information about the number of fecal samples tested for norovirus in HCT patients during this period was not possible to obtain. Two patients had two episodes of norovirus infection each, but we chose to include only the first episode for these patients. The median duration of symptoms was 9 (1–681) days. The demographical and clinical data in relation to secretor-status are presented in Table 1.

The number of secretor-negative patients was 7/89 (8%), which is a low proportion compared to the expected rate of at least 20% non-secretors present in the general Swedish population. Six of the secretor-negative patients had norovirus genogroup (G) II infection, and one had GI, but samples were not available for genotyping in these patients. The median norovirus Ct-value was 19.5 (8–23) in secretors and 30.5 (19–37) in non-secretors (*p* = 0.08), whereas the median duration of symptoms was 8 (1–681) and 16 (2–35) days in secretors and non-secretors, respectively (*p* = 0.38) (Table 1). Non-secretors were generally diagnosed with norovirus early after HCT (median 7 days) and had a significantly higher probability of concurrent mucositis compared to secretors (*p* = 0.03) (Table 1). Mucositis, gastrointestinal GVHD and/or other gastrointestinal infections were present at the time of norovirus infection in 6/7 (86%) of the non-secretors and in 39/82 (48%) of the secretors.

Information on norovirus genogroup was obtained for 87/89 patients, showing GI in 7/87 (8%) and GII in 80/87 (92%) of the patients. Genotyping of the norovirus strain could be performed in 18 patients and demonstrated GII.4 in 12 patients, GII.2 in 2 patients, GII.1 in 1 patient, GI.4 in 1 patient and GI.3 in 2 patients.

The patients infected with norovirus GII.4 were compared to those infected with other norovirus genotypes (non-GII.4), including four patients with norovirus genogroup I where genotyping was not possible (Table 2). The median duration of symptoms was 36 (3–681) days in patients with norovirus GII.4 and 15 (1–94) days in patients with non-GII.4 (*p* = 0.1). The median Ct-value was 18 (15–24) in cases of norovirus GII.4 and 20 (17–33) in non GII.4 (*p* = 0.06). All patients identified with norovirus GII.4 were secretor-positive. Additional data, including transplant procedures, are shown in an extended Table S1, supplementary material.

Eighteen of eighty-nine (20%) patients had chronic symptoms (>30 days) of norovirus infection. Genotyping was possible in 8/18 of these patients, showing GII.4 in six patients, GI.3 in one patient and GII.1 in one patient. Chronic norovirus symptoms were associated with SCID diagnosis (OR 10.7, 95% CI 1.8–62.1, *p* = 0.01) and concurrent gastrointestinal GVHD (OR 11.0, 95% CI 2.4–50.4, *p* <0.001) (Table 3). There was no relation between chronic norovirus symptoms and absolute lymphocyte count (ALC) <0.2 × 10^9^ mmol/L or absolute neutrophil count (ANC) <0.5 × 10^9^ mmol/L measured at 0, 14 or 30 days after diagnosis of norovirus infection (data not shown). Additional data comparing patients with chronic and non-chronic symptoms of norovirus are presented in an enlarged Table S2, supplementary material.

## 4. Discussion

Secretor-status is an important predictor of susceptibility to norovirus in immunocompetent individuals in a genotype-dependent manner. However, the role of secretor-status in the susceptibility to norovirus infection in immunocompromised patients is unclear [8].

We studied a cohort of HCT recipients diagnosed with norovirus infection. Only 8% were secretor-negative, which is a small proportion compared to the general population, suggesting that secretor-status affects the susceptibility to norovirus infection, even when the immune system is severely compromised. We have no data on secretor-status in HCT patients without norovirus infection. Still, with the wide variety of diagnoses as an indication for HCT, it is likely that the distribution of secretor-status reflects that of the normal population. Non-secretors had a median symptom duration of 16 days compared to 8 days in secretors. The number of non-secretors was, however, small and 6/7 of them had other potential causes of the gastrointestinal symptoms. Most non-secretors had high norovirus Ct-values (Table 1), further indicating other causes of gastrointestinal symptoms in these patients.

The norovirus genotype could not be determined in any of the secretor-negative patients. This is a limitation since the importance of secretor-status for norovirus susceptibility is strongly connected to the norovirus genotype [8]. Therefore, we could not exclude the possibility that norovirus GII.4 can infect secretor-negative immunocompromised patients, which with few exceptions does not occur in immunocompetent individuals [8]. Nevertheless, all (*n* = 12) norovirus genotype GII.4 strains in this study were identified in secretor-positive patients.

Previous data suggest that norovirus GII.4 is associated with a more severe clinical picture compared to other genotypes in immunocompetent children [14,15,16]. The importance of the norovirus genotype in relation to the clinical picture has not been previously reported in immunocompromised patients. We compared infections with norovirus genotypes GII.4 and non GII.4 and found possible differences in symptom duration between these groups, indicating that the norovirus genotype may have importance for the clinical course also in immunocompromised individuals. Yet, as the number of patients with an identified norovirus genotype was relatively small, the results should be interpreted with caution. Other factors such as concurrent gastrointestinal GVHD, mucositis, or gastrointestinal infection may also have affected the clinical course in some patients. The median Ct-value in patients with norovirus GII.4 was lower compared to non-GII.4 genotypes. A limitation is that three different qPCR methods were used, and the Ct values obtained may not be directly comparable between different methods. These results should thereby be interpreted carefully.

Chronic symptoms of norovirus in HCT patients have been linked to low counts of T-lymphocytes [28] and HCT performed due to a diagnosis of SCID [17], but factors associated with chronic norovirus infection in these patients remain poorly understood. SCID as the indication for HCT was associated with chronic norovirus symptoms in the present study, in line with our previous report [17]. Patients with SCID commonly have impaired and delayed immune reconstitution following HCT, indicating that the duration of norovirus symptoms is associated with the immune status of the patient, although detailed immunological characteristics of the patients are lacking in this study. Gastrointestinal GVHD was also significantly associated with chronic symptoms of norovirus, but it was not always possible to determine if either or both conditions were causing the symptoms.

## 5. Conclusions

Our results suggest that secretor-status affects the susceptibility to norovirus infection also in severely immunocompromised individuals. Hence, susceptibility to norovirus infection may depend primarily on genetics rather than on immunological factors. When infected, however, HCT patients with an underlying SCID diagnosis are at risk for chronic symptoms of norovirus, supporting the importance of immune control for clearance of the infection. Furthermore, the norovirus genotype may be a risk factor for chronic symptoms of norovirus infection in immunocompromised patients.

## Figures and Tables

**Table 1:** Demographical and clinical data in relation to secretor status.

	All, *n* = 89	Secretor-Positive, *n* = 82	Secretor-Negative, *n* = 7	*p*-Value
Symptoms of norovirus days, median (range)	9 (1–681)	8 (1–681)	16 (2–35)	0.38
Symptoms >30 days, *n* (%)	18 (20)	16 (20)	2 (29)	0.63
Ct-value, median (range)	20 (8–38)	19.5 (8–38)	30.5 (19–37)	0.08
Norovirus diagnosis, days post HCT, median (range)	86 (0–1328), *n* = 78	88 (0–1328), *n* = 72	7 (0–222), *n* = 6	0.2
Age, years median (range)	20 (0.5–67)	20 (0.5–67)	11 (1–42)	1
Diagnosis, *n* (%)				
*Malignant*	61 (69)	56 (68)	5 (71)	1
*SCID*	8 (9)	7 (9)	1 (14)	0.5
*Other non-malignant*	20 (22)	19 (23)	1 (14)	1
Donor, *n* (%)				
*Sibling*	19 (21)	18 (22)	1 (14)	1
*URD*	52 (58)	47 (57)	5 (71)	0.69
*Haplo*	18 (20)	17 (21)	1 (14)	1
SC source, *n* (%)				
*BM*	32 (36)	29 (35)	3 (43)	0.7
*PBSC*	48 (54)	45 (55)	3 (43)	0.7
*CB*	9 (10)	8 (10)	1 (14)	0.54
Any GVHD, *n* (%)	32 (36)	28 (34)	4 (57)	0.25
GI GVHD, (%)	17 (19)	13 (16)	1 (14)	1
Mucositis, *n* (%)	31 (35)	24 (29)	5 (71)	0.03
Other GI infection, *n* (%)	19 (21)	17 (21)	2 (29)	0.64

Ct, cycle threshold; HCT, allogeneic hematopoietic stem cell transplantation; SCID, severe combined immunodeficiency; Sibling, HLA-identical sibling donor; URD, HLA-matched unrelated donor; Haplo, haploidentical donor; SC, stem cell; BM, bone marrow; PBSC, peripheral blood stem cells; CB, cord blood; GVHD, graft-versus-host disease; GI, gastrointestinal. The Ct-value (from the first fecal sample at the time of diagnosis) was available in 76 (70 secretors, and 6 non-secretors) patients. Any GVHD includes acute GVHD grade I–IV, and chronic GVHD. Mucositis refers to gastrointestinal mucositis that occurs after chemotherapy or radiation therapy. GVHD, mucositis and “other GI infection” are included only when the condition occurs concurrently with symptomatic norovirus infection. Norovirus days post HCT refers to the number of days from HCT to norovirus diagnosis, in patients diagnosed after HCT. Eleven of the patients (ten secretor-positive and one secretor-negative patient) were diagnosed with norovirus infection 1–77 days prior to HCT (the symptom duration is only included from the day of HCT in these patients).

**Table 2:** Clinical details of patients with norovirus GII.4 and non-GII.4.

	Norovirus GII.4, *n* = 12	Norovirus Non-GII.4, *n* = 10	*p*-Value
Symptoms of norovirus, days, median (range)	36 (3–681)	16 (1–94)	0.1
Ct-value, median (range)	17.5 (15–24)	20 (17–33)	0.06
Secretor +, *n* (%)	12 (100)	9 (90)	0.45
Any GVHD *n* (%)	8 (67)	2 (20)	0.04
GI GVHD, *n* (%)	6 (50)	1 (10)	0.07
Mucositis, *n* (%)	2 (17)	6 (60)	0.07
Other GI infection, *n* (%)	2 (17)	2 (20)	1.0

Ct, cycle threshold; Secretor +, secretor-positive; GVHD, graft-versus-host disease; GI, gastrointestinal. Ct-value refers to the Ct-value from the first fecal sample at the time of diagnosis. Any GVHD includes acute GVHD grade I–IV, and chronic GVHD. Mucositis refers to gastrointestinal mucositis that occurs after chemotherapy or radiation therapy. GVHD, mucositis and “other GI infection” are included only when the condition occurs at the time of symptomatic norovirus infection. The group with non-GII.4 included six patients with identified genotypes other than GII.4, and four patients with genogroup I.

**Table 3:** Characteristics of the patients in relation to the symptom duration.

	Symptoms 1–30 d, *n* = 71	Symptoms > 30 d, *n* = 18	OR (95% CI)
Age, years median (range)	20 (1–67)	8.5 (0.5–65)	1.0 (0.98–1.03) *p* = 0.68
Diagnosis, *n* (%)			
*Malignant*	51 (72)	10 (55)	0.6 (0.2–1.6) *p* = 0.26
*SCID*	2 (3)	6 (33)	15.7 (2.8–86.5) *p* < 0.001
*Other non-malignant*	18 (25)	2 (11)	Ref
Norovirus, days post HCT, median (range)	88 (0–1328) (*n* = 64)	72 (0–276) (*n* = 14)	n.a.
Any GVHD, *n* (%)	21 (30)	11 (61)	4.3 (1.5–12.4) *p* = 0.01
GI GVHD, *n* (%)	9 (13)	8 (44)	7.0 (2.2–22.3) *p* < 0.001
Mucositis, *n* (%)	25 (35)	6 (33)	0.9 (0.3–2.8) *p* = 0.92
Other GI infection, *n* (%)	13 (18)	6 (33)	2.0 (0.6–6.3) *p* = 0.23

SCID, severe combined immunodeficiency; HCT, allogeneic hematopoietic stem cell transplantation; GVHD, graft-versus-host disease; GI, gastrointestinal; ref, reference; n.a.; not analyzed. Norovirus days post HCT refers to the number of days from HCT to norovirus diagnosis. Eleven of the patients (seven with symptoms 1–30 days and four with symptoms > 30 days) were diagnosed with norovirus infection 1–77 days prior to HCT. The symptom duration is only included from the day of HCT in these patients. Any GVHD includes acute GVHD grade I–IV, and chronic GVHD. Mucositis refers to gastrointestinal mucositis that occurs after chemotherapy or radiation therapy. GVHD, mucositis and “other GI infection” are included only when the condition occurs at the time of symptomatic norovirus infection.

## Data Availability

All data in the present study are available upon request to the authors within reason.

## Supplementary Materials

The following supporting information can be downloaded at: https://www.mdpi.com/article/10.3390/v14071350/s1, Table S1: Characteristics of patients with norovirus GII.4 compared with non-GII. 4, Table S2: Detailed characteristics of the patients with norovirus infection in relation to the symptom duration.
