# Supplementary material for: The Importance of Secretor-Status in Norovirus Infection Following Allogeneic Hematopoietic Stem Cell Transplantation
Source: Viruses. 2022 Jun 21;14(7):1350. doi: 10.3390/v14071350 (PMC9318794; doi:10.3390/v14071350)
Supplement: Supplementary file 1 [file viruses-14-01350-s001.zip › viruses-1715706-supplementary.pdf]

Supplementary material, methods

Sequencing and phylogenetic typing

RNA was extracted from norovirus positive feces using QIAamp Viral RNA Mini kit (Qiagen) according to manufacturer's instructions. 28 µl of RNA was subsequently mixed with 2.5 µg of random hexadeoxynucleotides [pd(N)6] (GE Healthcare, Uppsala, Sweden) and added to one RT-PCR bead (GE Healthcare, Uppsala, Sweden) with RNase-free water to a final volume of 50 µl. The RT reaction was carried out for 40 min at 42°C for cDNA synthesis. Amplification of a partial fragment of the N-terminal and shell region of the capsid was performed with a semi-nested PCR. For genogroup I, a first round PCR was performed using forward primer NVG1f1b [1] and reverse primer G1SKR[2], and a second round PCR generating a 330 bp fragment was performed using forward primer G1SKF [2] and reverse primer G1SKR. For genogroup II, a first round PCR was performed using forward primer QNIF2[3] and reverse primer G2SKR [2] and a second round PCR generating a 344bp fragment was performed using forward primer G2SKF[2] and reverse primer G2SKR. The PCRs were performed under the following conditions: 94°C for 4 min followed by 40 cycles of 94°C for 30 sec, 50°C for 30 sec, and 72°C for 1 min, with a final extension step at 72°C for 7 min. The amplicons were visualized on 2% agarose gel subsequently sent to Macrogen (Macrogen Inc, the Netherlands) for Sanger sequencing. The norovirus strains were later genotyped using an online norovirus genotyping tool (Norovirus Typing Tool Version 2.0, https://www.rivm.nl/mpf/typingtool/norovirus/) and with phylogenetic analysis using the MEGA software including reference strains of different genotypes.

References

1. Nordgren J, Bucardo F, Dienus O, Svensson L, Lindgren PE. Novel light-upon-extension real-time PCR assays for detection and quantification of genogroup I and II noroviruses in clinical specimens. *Journal of clinical microbiology* 2008; **46**(1)**:** 164-170. e-pub ahead of print 2007/10/26; doi: 10.1128/jcm.01316-07

2. Kojima S, Kageyama T, Fukushi S, Hoshino FB, Shinohara M, Uchida K *et al.* Genogroup-specific PCR primers for detection of Norwalk-like viruses. *Journal of virological methods* 2002; **100**(1-2)**:** 107-114. e-pub ahead of print 2001/12/18; doi: 10.1016/s0166-0934(01)00404-9

3. Loisy F, Atmar RL, Guillon P, Le Cann P, Pommepuy M, Le Guyader FS. Real-time RT-PCR for norovirus screening in shellfish. *Journal of virological methods* 2005; **123**(1)**:** 1-7. e-pub ahead of print 2004/12/08; doi: 10.1016/j.jviromet.2004.08.023

Table S1. Characteristics of patients with norovirus GII.4 compared with non GII.4

	GII.4, n= 12	non GII.4, n= 10
Symptoms of NV, days median (range)	36 (3-681)	15 (1-94)
Symptoms > 30 days, n (%)	6 (50)	2 (20)
Ct-value, median (range)	17.5 (15-24)	20 (17-33)
Norovirus, days post HCT, median (range)	109 (18-196)	95 (2-220)
Age, years median (range)	16 (1-63)	10.5 (1-44)
Gender male, n (%)	9 (75)	5 (50)
Sekretor + n (%)	12 (100)	9 (90)
Diagnosis, n (%)		
Malignant	7 (58)	9 (90)
SCID	3 (25)	0 (0)
Other non-malignant	2 (17)	1 (10)
Donor, n (%)		
Sibling	0 (0)	2 (20)
URD	9 (75)	5 (50)
Haplo	3 (25)	3 (30)
SC source, n (%)		
BM	3 (25)	6 (60)
PBSC	6 (50)	3 (30)
CB	3 (25)	1 (10)
RIC, n (%)	8 (67)	1 (10)
ATG/Alemtuzumab, n (%)	12 (100)	8 (80)
Any GVHD n (%)	8 (67)	2 (20)
aGVHD I-IV n (%)	8 (67)	2 (20)

aGVHD II-IV, n (%)	6 (50)	1 (10)
GI GVHD, n (%)	6 (50)	1 (10)
Mucositis, n (%)	2 (17)	6 (60)
Other GI Infection, n (%)	2 (17)	2 (20)

Ct, cycle threshold; secretor+, secretor positive; SCID, severe combined immunodeficiency; Sibling, HLA-identical sibling donor; URD, HLA-matched unrelated donor; Haplo, haplo-identical donor; SC, stem cell; BM, bone marrow; PBSC, peripheral blood stem cells; CB, cord blood; RIC, reduced-intensity conditioning; ATG, antithymocyte globuline; HCT, allogeneic hematopoietic stem cell transplantation; a, acute; GVHD, graft-versus-host disease; GI, gastrointestinal.

Norovirus days post HCT refers to the number of days from HCT to norovirus diagnosis. Six of the patients were diagnosed with norovirus on day -1 to -77 prior to HCT (two patients with GII.4 and four patients with non GII4). The symptom duration is only included from the day of HCT in these patients.

Mucositis refers to the gastrointestinal mucositis occurring after chemotherapy or radiation therapy. GVHD, mucositis and “other infection” are included only when the condition occurs at the time of symptomatic norovirus infection

Table S2. Detailed characteristics of the patients with norovirus infection in relation to the symptom duration

	Symptoms 1-30 d, n= 71	Symptoms > 30 d, n= 18	OR (CI) *p*
Age, years median (range)	20 (1-67)	8.5 (0.5-65)	1.0 (0.98-1.03) *p*=0.68
Diagnosis, n (%)			
Malignant	51 (72)	10 (55)	0.6 (0.2-1.6) *p*=0.26
SCID	2 (3)	6 (33)	15.7 (2.8-86.5) *p*<0.001
Other non-malignant	18 (25)	2 (11)	ref
Donor, n (%)			
Sibling	17 (24)	2 (11)	0.4 (0.1-1.8) *p*=0.21
Haplo	13 (18)	5 (28)	2.6 (0.4-17.0) *p*=0.31
URD	41 (58)	11 (61)	ref
SC source, n (%)			
BM	26 (37)	6 (33)	0.8 (0.3-2.3) *p*=0.65
CB	5 (7)	4 (22)	3.5 (0.8-14.5)

			p=0.09
PBSC	40 (56)	8 (44)	ref
RIC, n (%)	28 (39)	13 (72)	4.5 (1.4-13.8) *p*=0.01
ATG/Alemtuzumab, n (%)	56 (79)	16 (89)	2.3 (0.5-11.2) *p*=0.29
NV diagnosis, days post HCT, median (range)	88 (0-1328)	72 (0-276)	n.a.
Ct-value, median (range)	20 (12-38) n=54	19 (8-29) n=17	n.a.
Any GVHD, n (%)	21 (30)	11 (61)	4.3 (1.5-12.4) *p*=0.01
GI GVHD, n (%)	9 (13)	8 (44)	7.0 (2.2-22.3) *p*<0.001
Concurrent mucositis, n (%)	25 (35)	6 (33)	0.9 (0.3-2.8) *p*=0.92
Concurrent GI infection, n (%)	13 (18)	6 (33)	2.0 (0.6-6.3) *p*=0.23

Ct, cycle threshold; SCID, severe combined immunodeficiency; Sibling, HLA-identical sibling donor; URD, HLA-matched unrelated donor; Haplo, haplo-identical donor; SC, stem cell; BM, bone marrow; PBSC, peripheral blood stem cells; CB, cord blood; GVHD, graft-versus-host disease; GI, gastrointestinal; ref, reference; n.a., not analyzed

The Ct-value (from the first fecal sample at the time of diagnosis) was available in 76 (70 secretors, and 6 non-secretors) patients.

Any GVHD includes acute GVHD grade I-IV, and chronic GVHD. Mucositis refers to the gastrointestinal mucositis occurring after chemotherapy or radiation therapy. GVHD, mucositis and "other GI infection" are included only when the condition occurs concurrent with symptomatic norovirus infection

Norovirus days post HCT refers to the number of days from HCT to norovirus diagnosis, in patients diagnosed after HCT. Eleven of the patients (seven with symptoms 1-30 days and four with symptoms > 30 days) were diagnosed with norovirus infection 1-77 days prior to HCT (the symptom duration is only included from the day of HCT in these patients).
